# Elective Nodal Irradiation for Oligorecurrent Nodal Prostate Cancer: Interobserver Variability in the PEACE V-STORM Randomized Phase 2 Trial

**DOI:** 10.1016/j.adro.2023.101290

**Published:** 2023-06-13

**Authors:** Orane Lorton, Vérane Achard, Nikolaos Koutsouvelis, Maud Jaccard, Frederik Vanhoutte, Giovanna Dipasquale, Piet Ost, Thomas Zilli

**Affiliations:** a Department of Radiology, Geneva University Hospital, Geneva, Switzerland; b Department of Radiation Oncology, Fribourg Cantonal Hospital, Fribourg, Switzerland; c Department of Radiation Oncology, Geneva University Hospital, Geneva, Switzerland; d Faculty of Medicine, University of Geneva, Geneva, Switzerland; e Department of Radiation Oncology, Ghent University Hospital, Ghent, Belgium; f Iridium Network, Radiation Oncology, Antwerp, Belgium; g Department of Radiation Oncology, Oncology Institute of Southern Switzerland, EOC, Bellinzona, Switzerland; h Facoltà di Scienze Biomediche, Università della Svizzera Italiana, Lugano, Switzerland

## Abstract

**Purpose:**

Consistency in delineation of pelvic lymph node regions for prostate cancer elective nodal radiation therapy is still challenging despite current guidelines. The aim of this study was to evaluate the interobserver variability in elective lymph node delineation in the PEACE V **-** STORM randomized phase 2 trial for oligorecurrent nodal prostate cancer.

**Methods and Materials:**

Twenty-three centers were asked to delineate the elective pelvic nodal clinical target volume (CTV) of a postoperative oligorecurrent nodal prostate cancer benchmark case using a modified Radiation Therapy Oncology Group (RTOG) 2009 template (upper limit at the L4/L5 interspace). Overall, intersection and overflow volumes, Dice coefficient, Hausdorff distance, and count maps merged with computed tomography images were analyzed.

**Results:**

The mean volume including the 23 nodal CTVs was 430.4 ± 64.1 cm^3^, larger than the modified RTOG 2009 CTV reference volume (386.1 cm^3^). The intersection common volume between the modified reference RTOG 2009 and the 23 nodal CTVs was estimated at 83.9%, whereas the overflow volume was 23.4%, mainly located at the level of the presacral and the upper limit of the L4/L5 interspace. The mean Dice coefficient was 0.79 ± 0.02, whereas the mean Hausdorff distance was 27 ± 4.4 mm.

**Conclusions:**

In salvage radiation therapy treatment of oligorecurrent nodal prostate cancer, variations in elective lymph node volume delineation were mainly observed in the presacral and common iliac areas. Routine implementation and diffusion of available contouring guidelines together with a constant evaluation and evidence-based updating are expected to further decrease the existing variability in pelvic node contouring.

## Introduction

Pelvic nodal recurrence is one of the most commonly observed sites of relapse of prostate cancer after curative treatment of the primary. Different radiation therapy (RT) strategies have been proposed for salvage treatment of nodal oligorecurrent patients, ranging from focal stereotactic body RT to superextended lymph node irradiation.^1^

The PEACE V **-** STORM is a randomized, multicenter, phase 2 trial aiming to investigate the best treatment option for patients with oligorecurrent nodal disease. From June 2018 to May 2021, 196 patients with oligorecurrent prostate cancer with up to 5 pelvic nodes were randomized 1:1 to metastasis-directed therapies (MDT) alone (arm A) or MDT with whole pelvis elective nodal irradiation (WPRT) (arm B) (both arms combined with 6 months of androgen deprivation therapy [ADT]).^2^^,^^3^ The primary endpoint of this phase 2 trial was metastasis-free survival.

To improve the reliability of the study by reducing the interobserver variability (IOV), the PEACE V **-** STORM trial integrated a dedicated RT quality assurance program with a mandatory benchmark case (BC). Twenty-four centers completed the quality assurance of the trial, and assessment of outlining and treatment planning through this BC was conducted.^4^ Structures and treatment planning were evaluated as unacceptable variation, acceptable variation, or per protocol according to the Global Harmonization Group Guidelines.^5^ Per protocol and acceptable variation in delineation/dosimetry of the BC were communicated to the centers, which were activated for patient's inclusion. In case of unacceptable variation in delineation/dosimetry of the BC, centers were asked to submit new volumes/planning treatment with the requested changes. As regards to the delineation variations, the highest protocol deviations were observed for arm B with up to 46% of protocol deviations, whereas for the dosimetric variations, the greatest disparities were observed for arm A. Repeated BC resulted in an improved acceptable contouring and planning and adherence to the protocol.^4^

The fact that 46% of protocol deviations were observed in elective lymph node delineation, despite a clear protocol guidance, merits some further considerations. Going beyond a simple evaluation of the WPRT delineation variations, the aim of the present study was to assess the IOV in elective nodal definition among the participating centers by identifying areas of consensus and controversy. Results from this analysis could deserve as a basis for generating hypotheses about treatment efficacy and/or toxicity in clinical situations requiring WPRT.

## Methods and Materials

### Ethics approval and consent to participate

Signatures of informed consent were obtained from all patients before inclusion in the study. This study was approved by the ethics committee of the Ghent University Hospital (EC/2018/0130) and for all participating centers. The study is registered on Clinicaltrials.gov (NCT03569241) and Swiss National Clinical Trials Portal (SNCTP000002947).

### Delineation

The selected BC was a patient treated with radical robot-assisted laparoscopic prostatectomy and pelvic lymphadenectomy for a prostate adenocarcinoma, Gleason 4+3, pT3b pN0 (0/6) R1 at the final pathology, who experienced biochemical relapse with 2 positive pelvic lymph nodes (1 right external iliac node and a right obturator lymph node) at the restaging choline positron emission tomography (PET)/computed tomography (CT). Anonymized Digital Imaging and Communications in Medicine images of the CT and PET/CT scan of this patient were sent to the participating centers with the instructions to delineate the volumes and to perform treatment planning for each arm according to the trial protocol.^4^ Different center-specific treatment planning systems were used for clinical target volume (CTV) delineation.

In the arm B of the PEACE V **-** STORM trial, patients were treated with MDT plus WPRT (45 Gy/25 fx to the elective nodes with a simultaneous integrated boost at 65 Gy/25 fx to the positive nodes). For patients treated in the WPRT arm, the definition of the elective pelvic nodal CTV was mainly based on the Radiation Therapy Oncology Group (RTOG) 2009 guidelines,^6^ with the exception of an upper limit extended to the L4/L5 interspace to include the common iliac stations, as suggested by Spratt et al.^7^ As described in the guidelines, the nodal CTV includes the distal common iliac, the presacral, the external iliac, the internal iliac, and the obturator lymph nodes. Inclusion of the major pelvic vessels with a 7-mm radial margin avoiding organs at risk like the bowel, bladder, bone, and muscle is recommended.

As part of the RT quality assurance program of the trial, 23 participating centers electronically submitted their Digital Imaging and Communications in Medicine-RT nodal WPRT CTV structures to the coordinating center. These contours were then centralized, specifically identified with a matricule number, and imported into the Eclipse treatment planning system (Varian Medical Systems Inc, Palo Alto, CA). They were then compared with a reference CTV delineated by 2 experienced radiation oncologists (V.A. and T.Z.) according to the trial guidelines described previously.

### Assessment of the variability

The CTV contours and volume information of all participating centers were extracted from the Eclipse treatment planning system. The contours were transferred to a Velocity Workstation (Varian Medical Systems) for the extraction of indices. Contour analyses were performed using the Sorensen-Dice similarity coefficient to evaluate the similarity between the CTVs and the modified RTOG 2009 CTV reference, and the Hausdorff distance was used to assess the overall maximal distance between the reference and each CTV.^8^^,^^9^ The CTVs were compared using the common volume percentage, representing the ratio of the reference volume covered by a given center, and the volume overflow percentage, representing the ratio of the given volume in excess compared with the reference volume, as defined in Table 1. The indices and mean and standard deviations (SD) are presented in Table 2. To implement a precise qualitative evaluation of controversial and consensual regions, a count map of all CTVs was generated using the Matlab R2021b software (MathWorks, Natick, MA), with each voxel value determined by the superposition of participating centers, who included the corresponding image voxel within their CTV. For the 23 participating centers, the maximum count was 23, including a specific color map assigned to each number between 0 and 23 (consensus and controversial areas in red-orange and blue-green colors, respectively). The modified RTOG 2009 CTV reference volume was added to the final image to visualize the agreement between the nodal CTV delineated by the participating centers and the reference.

**Table 1:** Indices used in the variability assessment between the reference and the centers

Coefficient	Formula	Optimal value
Dice coefficient^8^	$\frac{2*\;(V_{ref}\cap \;V_{n})}{V_{ref}+\;V_{n}}$	1
Hausdorff distance^9^	$h\left(V_{ref},\;V_{n}\right)=\;\text{ma}x_{r\;\epsilon \;Vref}\;\left\{\text{mi}n_{n\;\epsilon \;Vn}\;\left\{\|r\;-n\|\right\}\right\}$ $h\left(V_{n},\;V_{ref}\right)=\;\text{ma}x_{n\;\epsilon \;Vn}\;\left\{\text{mi}n_{r\;\epsilon \;Vref}\;\left\{\|n-r\|\right\}\right\}$ $\text{Hd}\;=\;\text{max}\{\left[h\left(V_{ref},\;V_{n}\right),\;\;h\left(V_{n},\;\;V_{ref}\right)\right]\}$	0 mm
Common volume	$\frac{(V_{ref}\cap \;V_{n})*100}{V_{ref}}$	100%
Volume overflow	$\frac{(V_{n}-\;V_{ref}\cap \;V_{n})*100}{V_{n}}$	0%

*Abbreviations:*$V_{n}$ = volume of the center n to be compared r ∈ $V_{ref},$ and n ∈ $V_{n};$

$V_{ref}\;$= volume of the reference.

**Table 2:** Coefficients evaluating the interobserver variability in the contouring of nodal CTVs by each center compared with the modified reference RTOG 2009-based CTV

Center number	Volume, cm^3^	Dice coefficient	Hausdorff distance, mm	Common volume, %	Volume overflow, %
CTV 1	453.8	0.81	28.0	88.5	24.7
CTV 2	609.7	0.75	19.3	97.5	38.2
CTV 3	405.2	0.83	24.1	85.2	18.9
CTV 4	469.2	0.77	33.0	85.4	29.7
CTV 5	456.2	0.79	25.5	86.5	26.8
CTV 6	484.5	0.81	27.3	91.6	27.0
CTV 7	498.3	0.80	23.8	91.3	29.2
CTV 8	342.1	0.85	12.8	80.0	9.7
CTV 9	383.3	0.79	30.4	79.1	20.3
CTV 10	393.5	0.82	32.5	82.9	18.7
CTV 11	454.3	0.79	30.7	86.2	26.8
CTV 12	314.6	0.75	28.2	68.5	16.0
CTV 13	302.3	0.76	29.6	67.3	14.0
CTV 14	429.5	0.83	30.6	88.4	20.6
CTV 15	350.5	0.80	18.9	76.5	15.7
CTV 16	579.6	0.73	30.6	91.6	39.0
CTV 17	393.5	0.80	26.7	80.8	20.7
CTV 18	466.5	0.78	21.8	86.0	28.8
CTV 19	408.9	0.84	36.5	86.4	18.4
CTV 20	338.4	0.79	37.0	74.3	15.2
CTV 21	416.6	0.82	24.4	84.8	21.4
CTV 22	379.7	0.78	26.1	77.4	21.3
CTV 23	568.2	0.76	23.1	93.8	36.2
CTV reference	386.1	-	-	-	-
Mean	430.4	0.79	27.0	83.9	23.4
Standard deviation	64.1	0.02	4.4	5.9	6.3
Minimum	302.3	0.73	12.8	67.3	9.7
Maximum	609.7	0.85	37.0	97.5	39.0

*Abbreviations:* CTV = clinical target volume; RTOG = Radiation Therapy Oncology Group.

## Results

WPRT contouring data sets of all 23 participating centers were included in the final analysis, with Table 2 reporting the individual quantitative IOV indices results obtained for each center. Compared with the reference CTV (386.1 cm^3^), the cumulative mean CTV was 44.3 cm^3^ larger (430.4 ± 64.1 cm^3^), ranging from 302.3 to 609.7 cm^3^. In 16 centers, the CTV was larger than the reference, and for 7 centers the volume was smaller. In 35% of the cases (8 centers), the volume was in the ±10% range compared with the reference. In the other 15 centers, the differential range was between 20% and 45% higher for 8 centers and more than 45% in 3 centers (number 2, 16, and 23). On the other hand, for 4 centers the delineate volume was 10% smaller than the reference. Looking at the mean overflow volume, 23.4% of CTV exceeded the modified RTOG 2009 CTV reference volume. On the other hand, only 83.9% of the reference CTV was covered by the centers on average (common volume). For the 23 centers, the mean Sorensen-Dice similarity coefficient was 0.79 (SD, 0.02), and the mean Hausdorff distance was 27.0 mm (SD, 4.4 mm).

**Figure 1:**
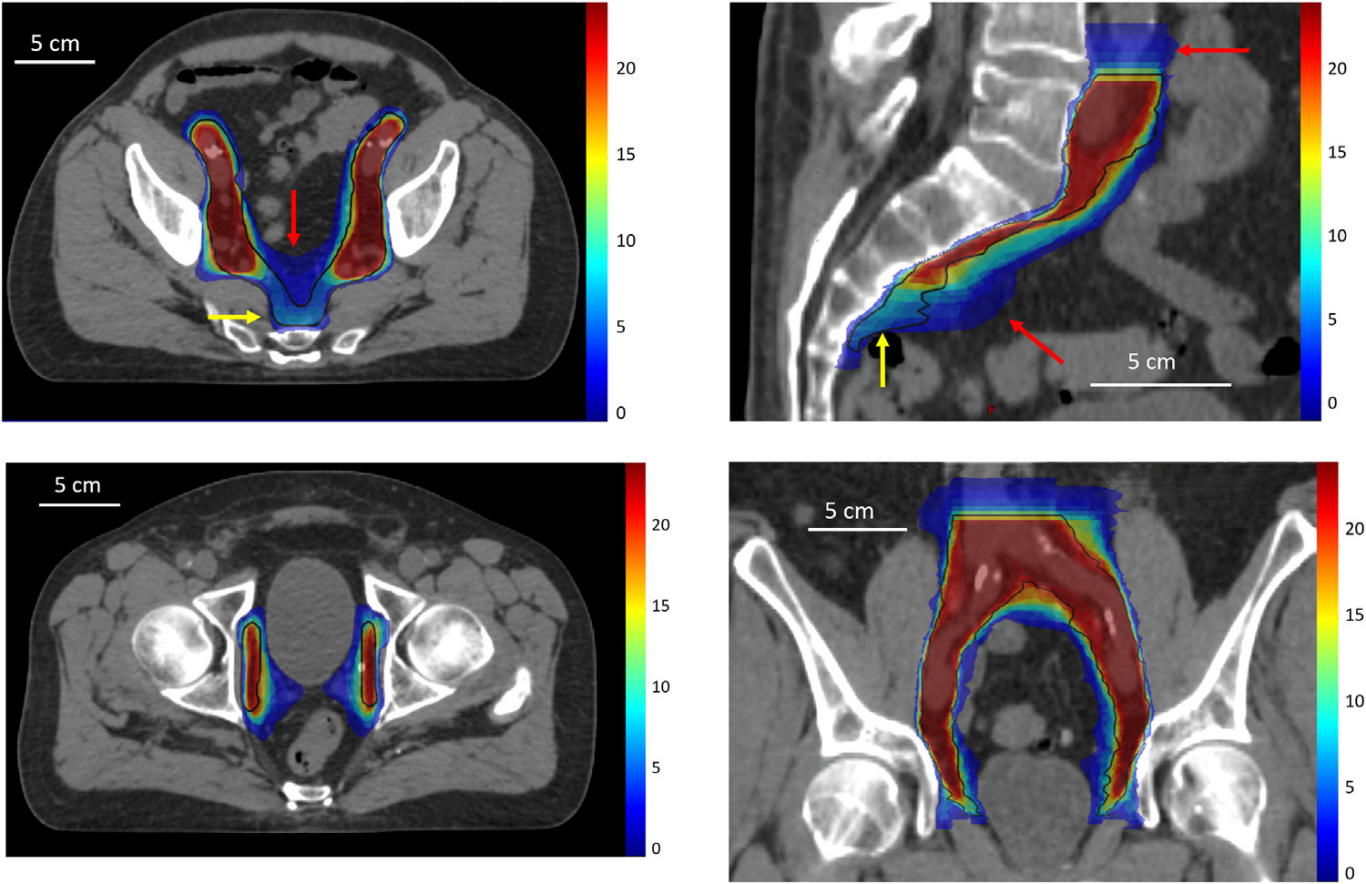
Count maps merged with planning computed tomography images illustrating the common and divergent areas in the axial (a) and sagittal (b) views. The color bar indicates the vote number of the voxels, the yellow arrows indicate the missing volume, and the red arrows indicate the exceeding volume compared with the reference clinical target volume (black contour). Axial view illustrating the lower contouring limit (c) and coronal view (d) showing the cranio-caudal extension of the clinical target volume.

From the count map (Fig. 1), 2 discordant contouring areas were identified compared with the reference modified-RTOG 2009 CTV: a larger CTV extending above the L4/L5 interspace and more anteriorly in the presacral area (Fig. 1a,b, red arrows) and a missing delineation volume in the caudal part of the presacral area at the level of S3 (Fig. 1a,b, yellow arrows). Table 3 reports the maximal distances between the modified RTOG 2009-based CTV and the nodal CTVs in the cranio-caudal directions for centers exceeding 2 mm in the upper and lower limit volume definition, according to the protocol guidelines. These deviations were observed in 6 centers in the upper limit (up to 17.5 mm) and in 5 in the definition of the lower border. For 2 centers (centers 15 and 5), these deviations were observed in both directions. Distances between the presacral border of the reference modified-RTOG 2009 CTV and the nodal CTVs in the sagittal plane are also presented in Table 3. In this area, 7 centers exceeded more than 2 mm and 2 presented a significant protocol deviation, ranging up to 25.7 mm anteriorly.

**Table 3:** Maximal distances between the modified RTOG 2009-based CTV and the nodal CTVs in the cranio-caudal direction for assessment of the upper and lower limit and in the sagittal direction for assessment of the presacral area

Center number	Upper limit: distance from the L4/L5 interspace, mm	Center number	Lower limit: distance from the superior pubic bone, mm	Center number	Anterior limit: distance from the presacral border, mm
CTV 22	17.5	CTV 15	5.6	CTV 23	25.7
CTV 16	14.0	CTV 5	5.6	CTV 7	22.6
CTV 5	12.1	CTV 3	4.1	CTV 4	15.5
CTV 9	8.1	CTV 17	4.1	CTV 2	12.9
CTV 23	4.2	CTV 2	4.1	CTV 5	10.6
CTV 15	4.2	Others	<1.9	CTV 21	8.3
Others	≤2.0			CTV 6	2.9
				Others	≤2.0

*Abbreviations:* CTV = clinical target volume; RTOG = Radiation Therapy Oncology Group.

## Discussion

Implementation of elective nodal RT in prostate cancer remains an active area of clinical investigation, with some emerging data suggesting better outcomes both in the primary^10^ and salvage settings.^11^ In the POP-RT randomized phase 3 trial, 224 patients with an estimated risk of nodal involvement over 20% (calculated using the Roach formula) were randomized to prostate-only RT or prostate plus WPRT. For pelvic nodal delineation, Murthy et al^10^ used a modified RTOG contour starting at L4-L5 interspace, as implemented in the PEACE V **-** STORM trial. After a median follow-up of 68 months, distant metastasis-free survival was significantly longer with WPRT compared with a prostate-only RT approach (95% vs 88%; *P* = .01).^10^ In the RTOG 0534 SPPORT trial, 1792 patients with persistent or rising prostate-specific antigen after radical prostatectomy were randomized to receive prostate bed (PB) RT alone (arm 1), PB plus short-term ADT (arm 2), or PB plus WPRT and short-term ADT (arm 3). The 5-year freedom from progression rate in the latter arm was superior to the 2 other arms (87.4% in arm 3 vs 70.9% and 81.3% in arms 1 and 2, respectively, *P* = .0001 for arms 2 and 3 *vs* arm 1).^11^ Pelvic nodal delineation was made according to the RTOG 2009 guidelines.^6^ Pending results of future trials, such as RTOG 0924 and PIVOTAL boost, will certainly help to provide additional level I evidence to the potential benefit of WPRT and consistency in the optimal delineation of pelvic nodal volumes.

When the PEACE V **-** STORM trial was conceived, the NRG 2020 guidelines^12^ were not yet published. To standardize contouring of elective nodal regions, the RTOG 2009 guidelines were selected as reference, with the exception of the upper level situated at the L4/L5 interspace based on the literature of the nodal recurrence patterns.^7^ Despite implementation of clear contouring guidelines, the present study highlights the existence of some degrees of variability in the delineation of the WPRT nodal regions, especially in the upper border limit and in the coverage of the presacral areas.

As proposed by Spratt et al,^7^ a cranial extension of the upper delineation limit of the nodal CTV at the L4/L5 interspace level should significantly improve the coverage of potential sites of failure. By extending the superior border at the L4/L5 interspace, the rate of pelvic recurrences receiving a full radiation dose increased from 41.7% when the superior border was located at L5/S1 interspace to 93.4%.^7^ In analogy, De Bruycker et al^13^ reported that 44% of the nodal recurrences detected by ^18^F-choline PET/CT imaging were encompassed by the CTV when the upper limit was at the level of L5/S1compared with a 73% rate when this limit was extended at the L4/5 interspace. Similar findings were reported by other series using prostate-specific membrane antigen (PSMA) PET tracers.^14^^,^^15^ Interestingly, in our study, the common iliac lymph node region was well covered by all participating centers, with some centers expanding their volumes even more cranially. Although both RTOG 2009^6^ and PIVOTAL^16^ templates used the L5/S1 as the superior delineation landmark, the recently published NRG 2020 guidelines recommend starting contouring at the bifurcation of the aorta into the common iliac arteries or the proximal inferior vena cava to the common iliac veins, whichever occurs more superiorly.^12^ Adequate coverage of this region may explain the better outcome results observed with the addition of WPRT in the POP-RT trial,^10^ whereas the GETUG-01^17^ and the RTOG 9413^18^ trials, which used an upper pelvic landmark around or below the L5/S1 interspace, showed no benefit from the addition of elective lymph node irradiation.^19^

As far as presacral nodes are concerned, only a few centers correctly covered this area despite inclusion of this region up to the S3 level being included in the RTOG 2009 guidelines.^6^ Using ^68^Ga-PSMA PET/CT for nodal mapping in patients with biochemical failure post radical prostatectomy, Devos et al^20^ identified up to 15% of positive presacral nodes. The fact that radiation oncologists tend not to cover this area could lead to lymph node misses and incorrect RT treatment. Our results are in agreement with Hall et al,^12^ who, using a count map strategy, identified the presacral area as one area of greatest variability.

One other important finding of our study is that the mean CTVs of the 23 centers were larger than the reference volume. Although the larger volume could potentially translate into an increased risk of toxicity, modern studies using intensity modulated or volumetric therapies showed very low rates of severe toxicity with WPRT.^21^, ^22^, ^23^, ^24^ In the RTOG 0534 SPPORT trial, 87.2% of patients were treated with intensity modulated RT and 12.8% with 3-dimensional conformal RT. Late toxicity was not significantly different between groups, apart from grade 2 or worse blood or bone marrow events in the group with PB plus WPRT compared with the group with PB only RT. The overall toxicity rate remained relatively low (5% vs 2%; *P* = .006).^11^

Results of the present study compare favorably with those reported by similar papers analyzing the IOV in pelvic lymph node delineation. Kiljunen et al^25^ evaluated IOV in delineating different structures in the pelvic region among 4 radiation oncologists and reported an average Dice coefficient of 0.76 for the lymph nodes compared with 0.79 in our study. Although the most effective educational intervention to improve contouring in RT is still unknown,^26^ Pasquier et al^27^^,^^28^ worked to improve homogeneity in target delineation of several tumor types, including prostate, head and neck, breast, brain, and lung cancer. In their study, target and organ-at-risk volumes delineated by senior radiation oncologists from 11 different institutions were initially compared using validated indexes and then reevaluated on new contours performed after discussion and harmonization of practices. The reported Dice coefficient was improved for the pelvic lymph node area from 0.58 to 0.63, although it still remains lower than the Dice coefficient calculated in our study.

As an effort to standardize pelvic lymph node volume definition, the NRG oncology consensus atlas on pelvic lymph node volumes for definitive and postoperative RT was recently published, implementing data on surgical mapping, lymphatic drainage series, and conventional/next-generation imaging studies on nodal recurrences.^12^ External validation of these guidelines was performed by Harmon et al^29^ and later by Vogel et al,^30^ who analyzed the nodal patterns of relapse of patients restaged with ^68^Ga-PSMA PET/CT and 18F-fluciclovine, respectively, for a biochemical relapse after radical prostatectomy. Compared with the RTOG 2009 guidelines, the updated NRG 2020-based CTV improved significantly the coverage of the pelvic lymph nodes. Nevertheless, even when using these updated contouring guidelines, up to 39% of the relapsing lymph nodes were not covered by the CTV in the Vogel et al study, highlighting the importance of using molecular imaging for restaging and definition of aberrant lymph node drainage stations. Interestingly, in our study the mean Sorensen-Dice similarity coefficient was higher (0.79; SD, 0.02) than the corresponding values observed in the Hall et al^12^ NRG study (ranging between 0.66-0.68). The presence of a larger contour set that included perirectal lymph nodes in the NRG study by Hall et al might explain this difference, with perirectal node areas being ultimately not included in the NRG 2020 guidelines. Finally, it is worth mentioning that the pursued goals differ between the 2 studies: Hall et al aimed to establish an international consensus atlas on pelvic lymph node volumes, whereas the aim of our study was to analyze the IOV in pelvic contouring based on a defined reference volume.

Limitations of the present work include the lack of a correlation between the volumes and the dosimetric data, including the potential effect on the surrounding organs at risk, though our previous work showed that despite the volume differences, doses constraints were respected for all plans.^4^ However, this study provides quantitative and qualitative analyses on real-world delineation of pelvic lymph node regions using specific contouring recommendations similar to the NRG 2020 guidelines.^12^

## Conclusion

In salvage RT treatment of oligorecurrent nodal prostate cancer, using a modified RTOG 2009 template, variations in elective lymph-node volume delineation were mainly observed in the presacral and common iliac areas, consistent with the NRG 2020 guidelines. Routine implementation and diffusion of available contouring guidelines together with constant evaluation and evidence-based updating are expected to further decrease the existing variability in pelvic node contouring.

## Disclosures

The authors declare that they have no known competing financial interests or personal relationships that could have appeared to influence the work reported in this paper.
